# The Acute Pharmacological Manipulation of Dopamine Receptors Modulates Judgment Bias in Japanese Quail

**DOI:** 10.3389/fphys.2022.883021

**Published:** 2022-05-11

**Authors:** Katarína Pichová, Ľubica Kubíková, Ľubor Košťál

**Affiliations:** Institute of Animal Biochemistry and Genetics, Centre of Biosciences, Slovak Academy of Sciences, Bratislava, Slovakia

**Keywords:** cognitive bias, spatial judgment task, decision-making under uncertainty, dopamine, Japanese quail

## Abstract

We have studied the effects of dopamine antagonists and agonists on Japanese quail behavior in the spatial judgment task. Twenty-four Japanese quail hens were trained in the spatial discrimination task to approach the feeder placed in the rewarded location (Go response, feeder containing mealworms) and to not approach the punished location (No-Go response, empty feeder plus aversive sound). In a subsequent spatial judgment task, the proportion of Go responses as well as approach latencies to rewarded, punished, and three ambiguous locations (near-positive, middle, near-negative, all neither rewarded nor punished) were assessed in 20 quail hens that successfully mastered the discrimination task. In Experiment 1, each bird received five treatments (0.1 and 1.0 mg/kg of dopamine D1 receptor antagonist SCH 23390, 0.05 and 0.5 mg/kg of dopamine D2 receptor antagonist haloperidol, and saline control) in a different order, according to a Latin square design. All drugs were administered intramuscularly 15 min before the spatial judgment test, with 2 days break between the treatments. Both antagonists caused a significant dose-dependent increase in the approach latencies as well as a decrease in the proportion of Go responses. In Experiment 2, with the design analogous to Experiment 1, the hens received again five treatments (1.0 and 10.0 mg/kg of dopamine D1 receptor agonist SKF 38393, 1.0 and 10.0 mg/kg of dopamine D2 receptor agonist bromocriptine, and saline control), applied intramuscularly 2 h before the test. The agonists did not have any significant effect on approach latencies and the proportion of Go responses in the spatial judgment task, as compared to the saline control, except for 10.0 mg/kg SKF 38393, which caused a decrease in the proportion of Go responses. The approach latency and the proportion of Go responses were affected by the cue location in both experiments. Our data suggest that the dopamine D1 and D2 receptor blockade leads to a decrease in the reward expectation and the negative judgment of stimuli. The effect of dopamine receptor activation is less clear. The results reveal that dopamine receptor manipulation alters the evaluation of the reward and punishment in the spatial judgment task.

## Introduction

The complex interactions between emotions and cognition offer new opportunities for the study of affective states (emotions) in animals. Emotions can influence cognitive processes by modifying attention, memory, or judgment in a short- or long-term manner. Cognitive processes can therefore provide a useful tool for the assessment of emotions in animals (Harding et al., 2004; Paul et al., 2005; Boissy et al., 2007; Mendl et al., 2009; Mendl and Paul, 2020).

Judgment bias tasks represent a group of tools for the assessment of the emotional states of animals based on decision-making under ambiguity (uncertainty). In this type of task, the judgment bias is estimated according to animals’ responses to ambiguous cues that have the spatial, visual, auditory, and/or olfactory attributes intermediate between the cues associated with positive and negative events (Harding et al., 2004; Roelofs et al., 2016). Biases in the judgment of ambiguous cues may provide a proxy measure of the valence of affective states in animals (Düpjan et al., 2013).

The spatial judgment task, originally introduced in rats (Burman et al., 2008), represents one of the alternatives how to test the judgment bias. In this task, animals are trained to expect a positive event in one location and a negative event in another. To determine whether animals are in a relatively positive or negative affective state, their response to ambiguous cues of intermediate spatial location is measured. Variations of this task have been used in many species, including birds (Košťál et al., 2020). The underlying mechanisms of the cognitive biases in animals (and man) are far from being understood. Many brain areas, such as the amygdala, prefrontal cortex, *nucleus accumbens*, ventral tegmental area, and various brain neurotransmitter systems, such as the dopamine, noradrenaline, opioid, and serotonin systems, appear to be involved in the processing of affective information (Mendl et al., 2009; Pessoa et al., 2019).

Principles from reinforcement learning theory can provide a model of the links between animal affect and decision-making (Mendl and Paul, 2020). Reinforcement learning is an adaptive process in which an animal utilizes its previous experience to improve the outcomes of future choices. The goal of reinforcement learning is to maximize future rewards. The reinforcement learning theories describe how the animal’s experience alters its value functions, which in turn influence subsequent decision-making (Lee et al., 2012). Activity related to reward expectancy has been found in many different brain areas. There is a lot of evidence supporting the idea that the phasic activity of dopaminergic neurons in the ventral tegmental area signals a discrepancy between the expected and actual reward outcomes (Schultz et al., 1997; Schultz, 2007). The reward prediction error signaling is not limited to the ventral tegmental area but has also been found in the prefrontal cortex (Oya et al., 2005), including the avian prefrontal cortex homolog (Packheiser et al., 2021).

Dopamine plays an essential role in reward in both birds and mammals, and it is found in analogous brain regions (Emery and Clayton, 2015; Durstewitz et al., 1999; von Eugen et al., 2020; Reiner et al., 2004). There is topographical, neurochemical, developmental, and hodological evidence in support of putative homologies of the mesolimbic reward system in vertebrates (O'Connell and Hofmann, 2011). Dopamine receptors D1A, D1B, and D1C called also D1D [see (Yamamoto et al., 2013)], belong to the D1 family, and the receptors D2, D3, and D4 belong to the D2 family (Kubikova et al., 2010; Kubikova and Košťál, 2010). The receptors D1A, D1B, and D2 are found in the avian brain highly expressed particularly in the striatum. The receptors D1C and D3 are expressed throughout the pallium and within the mesopallium, respectively, and the D4 receptors are found mainly in the cerebellum (Kubikova et al., 2010; Kubikova and Košťál, 2010). In red junglefowl chicks, it has been shown recently that individuals with the higher expression of dopamine D1 receptors in the prefrontal cortex are more optimistic (Boddington et al., 2020). Judgment bias was also related to the dopamine turnover rate in the mesencephalon of domestic chicks, with higher activity in individuals that had a more optimistic response (Zidar et al., 2018).

Pharmacological manipulations of affective states alter judgment bias [see (Neville et al., 2020) for review] although the evidence concerning the effects of dopaminergic drugs is sometimes controversial. Administration of the catecholamine precursor l-DOPA increased an optimism bias in humans (Sharot et al., 2009; Sharot et al., 2012). In contrast, the l-DOPA treatment of rats made them less likely to interpret ambiguous tone as a cue predicting reward, i.e., produced pessimism. This applied only to rats that were classified as “optimistic”, while the l-DOPA treatment did not affect the individuals classified as “pessimistic” (Golebiowska and Rygula, 2017). On the other hand, the acute administration of d-amphetamine induced positive bias in rats (Rygula et al., 2014a). Haloperidol, the D2 dopamine receptor antagonist, induced a negative bias in rats classified as “optimistic”, while it induced a positive bias in “pessimistic” rats (Golebiowska and Rygula, 2017).

This study aimed to test the hypotheses that blockade of dopamine receptors by antagonists induces a negative (pessimistic) bias while the activation of dopamine receptors by agonists induces a positive (optimistic) bias in Japanese quail. To test these assumptions we used spatial judgment task and pharmacological manipulation of the dopamine D1 and D2 receptors with selective antagonists and agonists. The choice of drugs was based on our previous study of kinetics and pharmacology of dopamine receptors in Japanese quail (Kubíková et al., 2009), as well as on the previous use of these drugs in avian behavioral studies (SCH 23390 (Akins et al., 2004; Schroeder and Riters, 2006), haloperidol (Moe et al., 2011; Moe et al., 2014), SKF 38393 (Balthazart et al., 1997; Balthazart et al., 1997) and bromocriptine (Kostal and Savory, 1994; Reddy et al., 2007)).

## Material and Methods

### Animals

The experiment was carried out using 24 adult Japanese quail (*Coturnix japonica*) hens. The animals were housed in wire cages with floor space 5620 cm^2^ in groups of maximally 12 quails per cage. Housing conditions were as follows: ambient temperature 20°C, relative humidity 55%, and light:dark cycle 16:8 h. The food (feeding mash for layers, 15% of crude protein, 11.5 MJ kg^−1^ metabolizable energy) and water were available *ad libitum* during the whole experiment. All procedures were approved by the State Veterinary and Food Administration of the Slovak Republic and by the Animal Ethics Committee of the institute.

### Testing Arena

**FIGURE 1:**
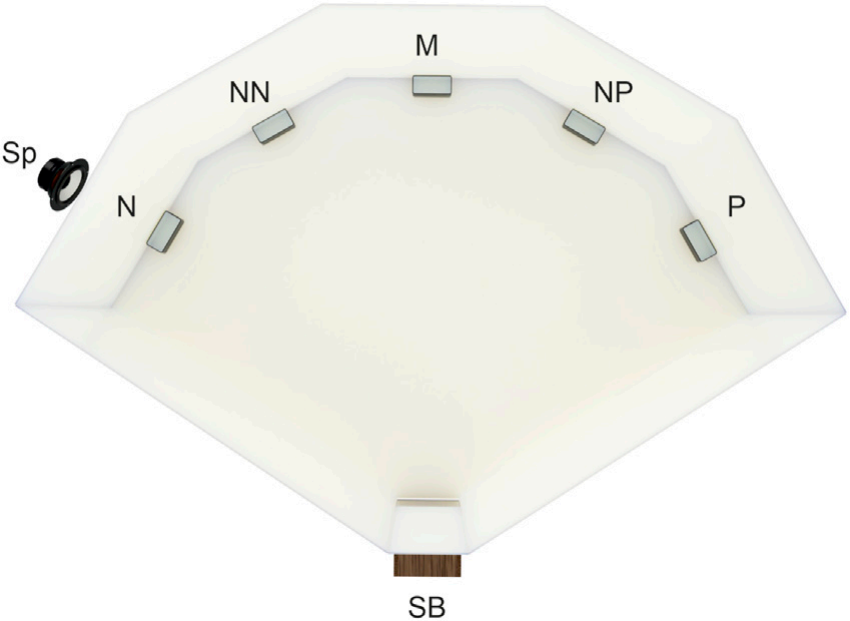
The experimental training/testing arena. Five possible feeder locations—positive (P, rewarded by the mealworms), negative (N, punished, empty feeder plus white noise *via* speaker Sp), and the three ambiguous locations (neither rewarded nor punished), near positive (NP), middle (M), and near negative (NN), start box (SB). For half of the quails, the positive location was on the right side of the test arena (as on the figure), and for the other half, it was on the left. During the training and testing, the feeder was placed at only one location per trial.

The method of judgment bias testing used in this study is a modification of the spatial judgment task introduced by Burman et al. (Burman et al., 2008) in rats. The testing arena was built from white plastic boards (PVC 800 mm × 400 mm × 3 mm; Figure 1). The wooden start box (150 mm × 150 mm × 150 mm) with a plexiglass guillotine door, that opened into the arena, was operated manually by the experimenter sitting out of the animals’ sight. The video camera (Microsoft LifeCam Cinema, Microsoft, United States) was placed above the arena allowing remote observation and recording of quail behavior. During training and testing, an opaque plastic cup feeder was placed close to the wall in one of the five locations 80 cm from the start box with a 40 cm distance between the neighboring locations. In any training or testing session, only one cup feeder was placed in one of the five possible locations.

The experiment consisted of the training phase, pharmacological manipulation, and testing phase. All animals were trained to consume mealworms used as a reward, habituated to the testing arena, and trained to associate one location with the reward and another location with the punishment. After this training, the animals were subjected to pharmacological manipulation using dopamine D1 and D2 receptor antagonists and agonists followed by the spatial judgment task (details described below).

### Habituation

During 3 days, the quails were habituated to mealworms as a food item, to the testing arena, and to transport from their home cage to the testing room in the start box. At this stage, the feeder containing a small amount of standard food and three mealworms were placed in the middle of the arena. Birds should leave the start box after opening the guillotine door, approach the feeder and consume the food. Each quail was subjected to one session with one trial per day for a maximum of 10 min.

### Positive Association Training

Birds were trained to associate the positive (P) feeder location with the reward (3 mealworms) for 4 days. This positive location was on the left side of the testing arena for half of the animals and on the right side of the arena for the rest of them. The birds were trained to leave the start box and enter the arena after opening the guillotine door, approach the feeder and consume the mealworms within 5 min. Each quail was subjected to one session per day. The first training session consisted of one trial while the next three sessions consisted of three trials. A trial was terminated once the animal reached the feeder and consumed the mealworms. In case the animal did not approach the feeder within the 60 s period, the maximum latency (60 s) was assigned for the trial. The interval between consecutive trials was 60 s. In each trial, the latency to approach the feeder (crossing the imaginary decision line 5 cm around the feeder) was recorded for each quail.

### Discrimination Task

**FIGURE 2:**
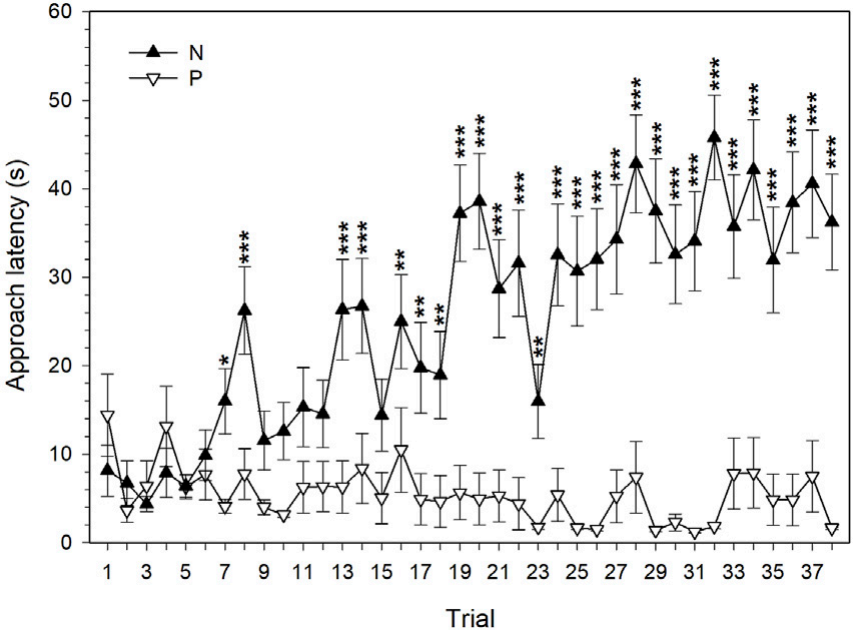
The latency to approach the rewarded positive (P) and punished negative (N) feeder locations (mean ± SEM) by quail hens (*n* = 20). *−*p* < 0.05, **−*p* < 0.01, ***−*p* < 0.001.

During the spatial discrimination task training, the quails were trained to associate the positive feeder location (P) with the reward and the negative feeder location (N) with the punishment. The quails were trained to approach the P location and consume mealworms and refrain from approaching the N location to avoid punishment (empty feeder and 5 s of 80 dB white noise). For half of the animals the P location was on the left side and the N location on the right side of the arena, while for the other half of the animals the positions of the feeders were reversed. Each bird was subjected to one training session per day. The first five training sessions consisted of four trials (2 P, 2 N) presented in random order, i.e., together 10 P and 10 N trials (Figure 2, trials 1–10). In the consecutive 14 sessions, the number of trials was increased to five. The initial trial of the session, not included in the analyses, was P, followed by four measured trials (2 P, 2 N) in random order, i.e., 28 P and 28 N trials (Figure 2, trials 11–38). The order of the feeder locations was the same for all animals within the training day but it differed in each session. For each animal, the latency to approach the feeder (crossing the imaginary decision line 5 cm around the feeder) after leaving the start box was recorded. In case the animal did not approach the feeder within this time, a trial was terminated, the animal was returned to the start box and the next trial started after 60 s. A correct response was defined as approaching the feeder (i.e., a Go response) within 60 s following a positive cue and not approaching the feeder (i.e., a No-Go response) within 60 s following a negative cue.

### Spatial Judgment Task

Four quails were excluded from further testing since they failed to discriminate between the P and N locations after the end of the discrimination training, i.e., the number of birds included in the judgment bias tests was reduced to 20. The spatial judgment task testing included the presentation of the feeder in P and N locations and also in three ambiguous intermediate spatial locations (NP—near positive, M—middle, NN—near negative; Figure 1) that were neither rewarded nor punished. The animals were subjected to one session after each pharmacological treatment (see Drug Treatment below). The sessions consisted of seven trials in total, starting with one trial with P and one N location to foster the learned discrimination (data from these two trials were not included in the analyses), and after that, the feeder was placed in all five locations in random order. The order of the locations differed between the sessions. Again, the latency to approach the feeder i. e., the Go response (crossing the imaginary decision line 5 cm around the feeder) within 60 s after leaving the start box was recorded. In case that animal did not approach the feeder within this time (No-Go response) a trial was terminated, the animal was returned to the start box and after the 60 s break, the next trial started.

### Drug Treatment

Dopaminergic drugs were selected based on their pharmacological properties and receptor binding in the quail brain (Kubíková et al., 2009). All drugs were obtained from RBI/Sigma-Aldrich (St. Louis, United States). Drug treatments were divided into two experiments, Experiment 1 using dopamine receptor antagonists SCH 23390 for D1 and haloperidol for D2 and Experiment 2 using dopamine receptor agonists SKF 38393 for D1 and bromocriptine for D2.

Experiment 1 included five treatments: 0.1 mg/kg of SCH 23390, 1 mg/kg of SCH 23390, 0.05 mg/kg of haloperidol, 0.5 mg/kg of haloperidol, and saline control. All solutions were injected intramuscularly in the amount of 1 ml/kg 15 min before the start of the spatial judgment task. Experiment 2 included five treatments: 1.0 mg/kg of SKF 38393, 10.0 mg/kg of SKF 38393, 1.0 mg/kg bromocriptine, 10.0 mg/kg bromocriptine, and the saline control. All solutions were injected intramuscularly in the amount of 1 ml/kg 2 h before the start of the spatial judgment task. The doses of drugs, their dissolving, and the timing of injection were based on published data (Kostal and Savory, 1994; Moe et al., 2014). The time-course of bromocriptine effects on the behavior of rats (Pizzolato et al., 1985) as well as chickens (Kostal and Savory, 1994) is biphasic, with initial suppression and delayed stimulation. Biphasic locomotor effects were also reported after the treatment with SKF 38393 in mice (Tirelli and Terry, 1993). Therefore the treatments in Experiment 2 were applied 2 h before the behavioral tests. In both experiments, all animals received all five treatments in a different order, according to a Latin square design. To minimize the carry-over effect, there was a 2-day break between each treatment.

### Data Analysis

Data were analyzed using the GLIMMIX procedure for generalized linear mixed models in SAS (version 9.04; SAS Institute Inc. Cary, NC, United States). Latency data from the spatial discrimination training were analyzed using the model with two fixed factors, feeder position (P, N) and trial. Latency data from Experiment 1 and Experiment 2 were analyzed with the feeder position (P, NP, M, NN, N), treatment, and their interaction as fixed effects. The proportions of the Go responses based on binary data (Go response = 1, No-Go response = 0) were also analyzed using the GLIMMIX procedure but with binomial distribution and logit link function. In all analyses, individual bird identity was taken into account as a random effect. Multiple post-hoc comparisons were done using the Tukey–Kramer test.

## Results

### Discrimination Learning

There was a significant effect of the feeder position (F_1,1425_ = 537.98, *p* < 0.001), trial (F_37,1452_ = 4.75, *p* < 0.001), and their interaction (F_37,1452_ = 6.25, *p* < 0.001) on the approach latency during the discrimination learning. The first significant difference between the latencies to approach the P and N cues during the discrimination training was observed in the 7th trial (Figure 2). Although there were fluctuations in the discrimination performance of quail hens, from the 16th trial on the mean latency to reach the feeder in the P location was significantly shorter than the latency to reach the feeder in the N location. Beginning from the 19th trial, the mean difference between the latencies to approach the P and N location of the feeder was, with one exception (the 23rd trial), more than 20 s. The maximum mean difference of 43.95 s between the latencies to reach the P and N location was observed in the 32nd trial (Figure 2).

### Spatial Judgment Task

**FIGURE 3:**
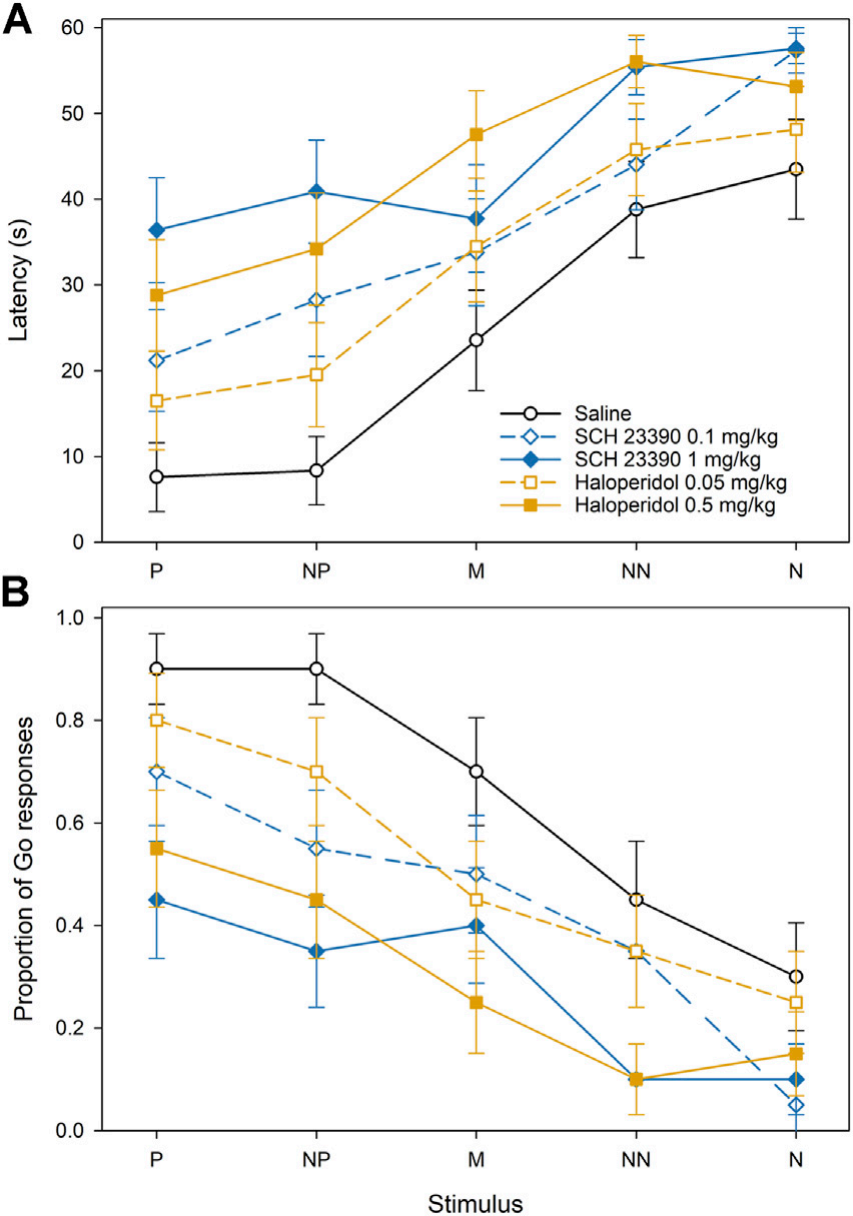
The effect of dopamine D1 and D2 receptor antagonists on the approach latencies **(A)** and a mean proportion of Go responses **(B)** of Japanese quail hens in the spatial judgment task. Mean ± SEM of the approach latencies to the positive (P), near-positive (NP), middle (M), near-negative (NN), and negative (N) cues.

In Experiment 1, there was a significant effect of the feeder position (F_4,456_ = 40.20, *p* < 0.001) and treatment (F_4,456_ = 17.54, *p* < 0.001), but not their interaction, on the approach latencies (Figure 3A). There were dose-dependent increases in approach latencies after the treatment with both antagonists, SCH 23390 (D1) as well as haloperidol (D2). The post-hoc comparisons showed that the approach latencies after the treatment with 0.1 mg/kg SCH 23390 were significantly longer in comparison with control (*p* < 0.001), while the latencies after the treatment with 1 mg/kg SCH 23390 latencies were significantly longer in comparison with treatment with 0.1 mg/kg SCH 23390 (*p* < 0.05) as well as with the saline control (*p* < 0.001). The post-hoc comparisons also showed that the approach latencies after the treatment with 0.05 mg/kg haloperidol were significantly longer in comparison with control (*p* < 0.05), and the approach latencies after the treatment with 0.5 mg/kg haloperidol were significantly longer in comparison with 0.05 mg/kg haloperidol (*p* < 0.01) as well as with the saline control (*p* < 0.001). The pattern of the proportions of the Go responses after the treatment with dopamine antagonists in Experiment 1 was reciprocal to latencies. There was a significant effect of the feeder position (F_4,456_ = 19.19, *p* < 0.001) and treatment (F_4,456_ = 12.25, *p* < 0.001), but not their interaction, on the proportions of the Go responses. The proportion of the Go responses decreased after the treatment with both, SCH 23390 (D1) and haloperidol (D2) (Figure 3B). The proportions of the Go responses after the treatment with 0.1 mg/kg as well as 1.0 mg/kg of SCH 23390 were significantly lower in comparison with the control (*p* < 0.001). There was no significant difference in the proportion of the Go responses between the two doses of SCH 23390. The proportions of the Go responses after the treatment with 0.05 mg/kg as well as 0.5 mg/kg of haloperidol were significantly lower in comparison with control (*p* < 0.05 and *p* < 0.001, respectively), and the proportion of Go responses after the treatment with 0.5 mg/kg of haloperidol was significantly lower in comparison with 0.05 mg/kg of haloperidol (*p* < 0.01).

Both, the approach latencies and the proportion of Go responses in Experiment 1 were affected by the cue location. The latency to approach the feeder in the P location was significantly shorter than the latency to approach the feeder in the M, NN, and N locations (all *p* < 0.001), the latency to approach the NP location was shorter than the latency to approach the M (*p* < 0.05), NN, and N (*p* < 0.001), and the latency to approach the M was significantly shorter than the NN and N (*p* < 0.001). The proportion of Go responses to the feeder in the P location was significantly higher than the proportion of the Go responses to the feeder in the M, NN, and N locations (all *p* < 0.001), the proportion of the Go responses to the NP location was higher than the proportion of the Go responses to NN, and N locations (*p* < 0.001), and the proportion of the Go responses to the feeder in the M location was significantly higher than the NN (*p* < 0.01) and N (*p* < 0.001).

**FIGURE 4:**
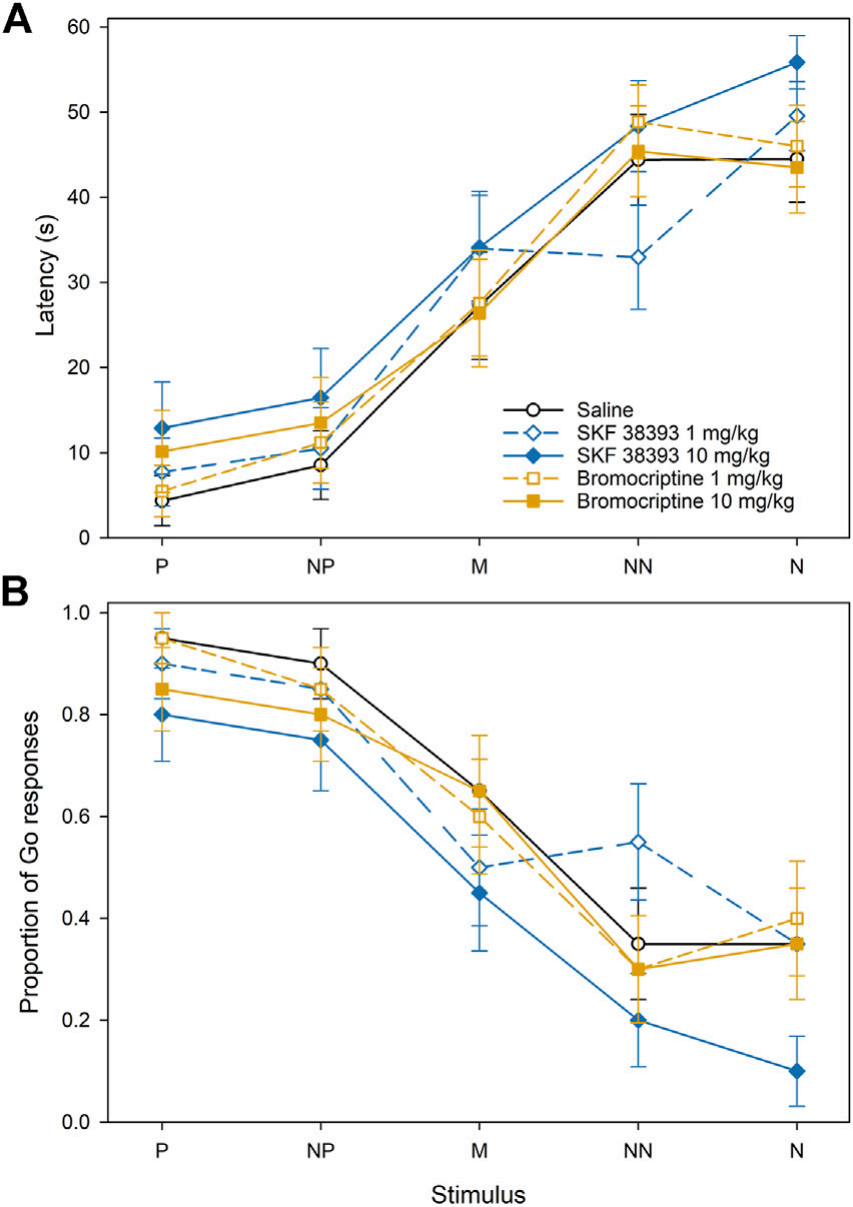
The effect of dopamine D1 and D2 receptor agonists on the approach latencies **(A)** and mean proportion of Go responses **(B)** of Japanese quail hens in the spatial judgment task. Mean ± SEM of the approach latencies to the positive (P), near-positive (NP), middle (M), near-negative (NN), and negative (N) cues.

In Experiment 2, there was a significant effect of the feeder position (F_4,456_ = 40.20, *p* < 0.001), but the effects of treatment and treatment × position interaction on the approach latencies were not significant (Figure 4A). However, in case of the proportion of the Go responses, there was a significant effect of both the feeder position (F_4,456_ = 20.15, *p* < 0.001) and the treatment (F_4,456_ = 3.83, *p* < 0.01). The post-hoc comparisons of treatments showed that the treatment with 10.0 mg/kg of SKF 38393 led to the significant decrease of the proportion of the Go responses in comparison with the control (*p* < 0.001).

The approach latencies and the proportion of the Go responses were affected by the cue location similarly to Experiment 1. The latency to approach the feeder in the P location was significantly shorter than the latency to approach the feeder in the M, NN, and N locations (all *p* < 0.001), the latency to approach the NP location was shorter than the latency to approach the M, NN, and N (all *p* < 0.001), and the latency to approach the M was shorter than the latency to approach the NN and N (all *p* < 0.001). The proportion of Go responses to the feeder in the P location was significantly higher than the proportion of the Go responses to the feeder in the M, NN, and N locations (all *p* < 0.001), the proportion of the Go responses to the NP location was higher than the proportion of the Go responses to M, NN, and N locations (all *p* < 0.001), and the proportion of the Go responses to the feeder in the M location was significantly higher than the NN and N (all *p* < 0.001).

## Discussion

In this study, we were testing the hypothesis that dopaminergic signaling affects decision-making under ambiguity. We were evaluating the response of Japanese quail hens in the spatial judgment task following the blockade or activation of specific dopamine receptors belonging to the D1 and D2 families. The dopamine receptor blockade led to increased response latencies and decreased proportion of the Go responses, indicating possible pessimistic bias. Although the dopamine receptor activation has not affected the latencies of the responses in the judgment bias task, the higher dose of the D1 agonist SKF 38393 decreased the proportion of the Go responses.

The cognitive bias paradigm used for the study of affective states evaluates biases in processing towards reaching a reward or avoiding punishment. Therefore the changes in circuits involved in reward and punishment are expected. Human and animal studies identified that the reward circuits are centered around the dopaminergic neurons in various brain regions (Hales et al., 2014). Dopamine is implicated in the valuation of reward-related cues (Schultz et al., 1997; Schultz, 2007). That is why the dopaminergic system is considered one of the candidate systems involved in the mechanisms underlying judgment bias (Mendl et al., 2009; Noworyta et al., 2021).

The distribution of the dopamine receptors in the avian brain (Kubikova et al., 2010) and their pharmacological properties (Kubíková et al., 2009) are similar to those in mammals. Boddington et al. (Boddington et al., 2020) linked the D1 receptors with optimistic judgment when they found the higher expression of dopamine D1 receptors in the prefrontal cortex of junglefowl chicks which had shorter latencies to approach the ambiguous cues. In the same study, the expression of the D2 receptors in the prefrontal cortex did not correlate significantly with the optimism in the judgment bias test.

Our main prediction in this study was that the treatment with dopamine receptor antagonists will induce the negative/pessimistic judgment bias (prolonged approach latencies and decreased proportion of the Go responses in response to ambiguous cues), while conversely, the treatment with dopamine receptor agonists will induce the positive/optimistic bias (shorter latencies, increased proportions of the Go responses). Blockade of dopamine receptors by antagonists SCH 23390 and haloperidol in our experiment prolonged the latencies to reach the feeders in ambiguous locations in the judgment bias tests and decreased the proportions of the Go responses. However, the effect was not restricted to ambiguous cues only. The latencies to reach the feeder in positive and negative (reference) locations were prolonged too. The same applies to the reduction in proportions of the Go responses. Similar “generalized response bias” was reported by Rygula et al. (Rygula et al., 2014b) in rats after the treatment with the norepinephrine–dopamine reuptake inhibitor mazindol. Rats showed overall decreased positive and overall increased negative responses in an operant Go/Go task generalized to the reference cues. The authors concluded that it may reflect a decreased expectation of reward and increased expectation of punishment, resulting from a change in the perception of the likelihood of receiving the reward and punishment, respectively, or a change in the value of the reinforcement. The combined treatment of rats with the norepinephrine reuptake inhibitor reboxetine and corticosterone had a similar effect. It also decreased the proportion of positive responses in an operant Go/Go task to all, ambiguous as well as both reference cues (Enkel et al., 2010; Anderson et al., 2012). Anderson et al. (Anderson et al., 2012) hypothesized that the treatment caused a reduction in motivation to respond for reward. Similarly, in chicks in the depression-like state, not only the latencies to reach the ambiguous cues, but also the latency to reach the aversive cue in the runway test were increased (Salmeto et al., 2011). More generally, the meta-analysis of pharmacological manipulations of judgment bias confirmed that such manipulations do not influence only the response to ambiguous cues, but also the response to reference cues, or more precisely, these manipulations exerted a similar effect at the negative reference cue compared with the probe cues (Neville et al., 2020). On the other side, in the seminal study of Harding et al. (2004), rats in a mild depression-like state were slower to press the lever and tended to show fewer responses in an operant Go/NoGo task not only in reaction to the ambiguous cues but also to the positive one.

If we accept the “generalized response bias” idea, then both dopamine receptor antagonists in our study resulted in a negative/pessimistic bias in Japanese quail judgment. Such results were found in several other studies. The acute haloperidol treatment in rats caused a “pessimistic” shift in “optimists”, but an “optimistic” shift in “pessimists” (Golebiowska and Rygula, 2017). Haloperidol had also a negative effect on spatial reference and working memory in the case of another type of spatial task in rats, the spatial cone field task (Blokland et al., 1998). Dopamine antagonist fluphenazine, with the affinity for the D2 site slightly greater than for the D1 (Morgan and Finch, 1986), abolished optimistic judgment in bumblebees after sucrose consumption (Mendl and Paul, 2016; Solvi et al., 2016). On the other hand, the acute haloperidol treatment has been shown to enhance dopamine turnover in the rat striatum (Lerner et al., 1977; Rastogi et al., 1982) as well as in the domestic chicken prefrontal cortex homolog (Gruss et al., 2003), but the increased dopamine turnover in the mesencephalon domestic fowl females was associated with a more optimistic response in a judgment bias test (Zidar et al., 2018). Microinfusion of the D1 receptor antagonist SCH 23390 into the *nidopallium caudolaterale*, a prefrontal cortex homolog of pigeon, caused an impairment of a visual discrimination reversal (Diekamp et al., 2000) but did not affect the performance in a delayed-matching-to-sample task (Herold et al., 2008). In our experiment, the blockade of D1 receptors caused a similar “generalized response bias” as the blockade of D2 receptors.

A possible explanation of the prolonged latencies to reach the feeder locations after the treatment with dopamine receptor antagonists could be sedation, changes in motor function, which can complicate interpretation of results (Bushnell and Levin, 1993). Haloperidol at 0.5 mg/kg for example decreased the response rate of pigeons in a delayed-matching-to-sample task but failed to impair accuracy (Poling et al., 1984). However, 0.5 mg haloperidol/kg body weight did not have any clear sedative effect in laying hens (Kjaer et al., 2004). Similar results were reported by Moe et al. (Moe et al., 2014), who found that the latency to walk to the reward and start to eat in laying hens was affected only by the high dose of haloperidol (2 mg/kg) but not by the 0.5 mg/kg dose. Zarrindast and Namdari (Zarrindast and Namdari, 1992), who used the highest dose of SCH 23390 in chickens corresponding to our lower dose state that SCH 23390 did not induce catalepsy or marked sedative effect.

The treatment with dopamine receptor agonists SKF 38393 and bromocriptine have not affected significantly the latencies to approach the cues by quail hens in the spatial judgment task. However, the acute treatment with the SKF 38393 at the dose of 10.0 mg/kg led to the decreased proportions of Go responses. The published data on the influence of drugs with agonistic effects on the dopaminergic system on the judgment bias are inconsistent. Although the l-DOPA induced optimistic bias in humans (Sharot et al., 2009; Sharot et al., 2012), it failed to induce optimism in rats, where the acute administration of l-DOPA induced “pessimism” in animals classified as “optimistic” and did not affect the judgment bias of “pessimistic” animals (Golebiowska and Rygula, 2017). The indirect dopamine agonist d-amphetamine induced optimistic bias in rats (Rygula et al., 2014a). On the other hand, the lack of effect of the SKF 38393 on judgment bias in quail can be maybe attributed to the fact that it is a partial D1 dopamine receptor agonist. It has been shown in rats that it has a lower affinity for the specific dopamine receptors than the full D1 receptor agonists and also its effects on learning and memory differ (Watts et al., 1993; Amico et al., 2007).

Behavioral effects of SCH 23390 and SKF 38393 do not always correspond to their established receptor subtype selectivity at D1 receptors (Zarrindast et al., 2011). For example, it was shown that both SKF 38393 and SCH 23390 induce inhibition of feeding in rats (Zarrindast et al., 1991). In our experiment, both SCH 23390 and SKF 38393 (at the high dose) decreased the proportion of Go responses of quail in the spatial judgment task. Terry and Katz (Terry and Katz, 1994) called into question the use of SKF 38393 as a D1 agonist in studies of feeding, and other contexts as well. Inhibition of feeding in rats by SKF 38393 and SCH 23390 can be according to some studies mediated *via* serotonergic mechanisms (Zarrindast et al., 1991; Zarrindast et al., 2011), that also play a role in a judgment bias (Golebiowska and Rygula, 2017; Neville et al., 2020).

Affective states differ in their duration. Mood differs from shorter-term (acute) emotions in that it represents a state that usually lasts over a longer period of time such as days or weeks (Mendl et al., 2010; Webb et al., 2019). A recent theory argues that mood reflects the cumulative impact of differences between obtained outcomes and expectations. The judgment bias tests were developed to assess mood (Raoult et al., 2017). That points out to the possible importance of duration of treatment in case of pharmacological induction of affective states. The acute and chronic pharmacological treatments influence them in a different ways. The study investigating the affect-induced cognitive bias in rats using systemic treatments with anxiolytic (diazepam) and antidepressant drugs (reboxetine or fluoxetine) suggested that judgment bias may be sensitive to chronic but not acute antidepressant treatment (Anderson et al., 2012). Hales et al. (Hales et al., 2017) state that experiments to date have generally failed to observe consistent effects with conventional antidepressants following acute administration. That can be also a possible explanation of the minor effects of the dopamine agonists used in this study. The acute treatment might not induce large enough differences in the affective state detectable by the spatial judgment task.

## Conclusions

The inhibition of dopamine D1 and D2 receptors by SCH 23390 and haloperidol led to prolonged latencies to approach the feeder in ambiguous locations in the spatial judgment task, but also latencies to approach positive and negative locations. It also decreased the proportions of Go responses. This suggests the induction of negative, pessimistic judgment bias, but also a generally lower expectation of the reward. On the other hand, the activation of dopamine D1 and D2 receptors by SKF 38393 and bromocriptine did not have any significant effect on the spatial judgment task latencies within the range of doses applied. However, the larger dose of the SKF 38393 decreased the proportion of the Go responses. The present results demonstrate that dopamine signaling is involved in the underlying mechanisms of judgment bias in Japanese quail.

## Data Availability

The raw data supporting the conclusions of this article will be made available by the authors, without undue reservation.
